# *e*MIFS: A Normalized Hyperbolic Ransomware Deterrence Model Yielding Greater Accuracy and Overall Performance

**DOI:** 10.3390/s24061728

**Published:** 2024-03-07

**Authors:** Abdullah Alqahtani, Frederick T. Sheldon

**Affiliations:** 1 College of Computer Science and Information Systems, Najran University, Najran 61441, Saudi Arabia; 2 Department of Computer Science, University of Idaho, Moscow, ID 83844, USA; sheldon@uidaho.edu

**Keywords:** ransomware, cyber security, feature selection, MIFS, crypto-ransomware, early detection

## Abstract

Early detection of ransomware attacks is critical for minimizing the potential damage caused by these malicious attacks. Feature selection plays a significant role in the development of an efficient and accurate ransomware early detection model. In this paper, we propose an enhanced Mutual Information Feature Selection (*e*MIFS) technique that incorporates a normalized hyperbolic function for ransomware early detection models. The normalized hyperbolic function is utilized to address the challenge of perceiving common characteristics among features, particularly when there are insufficient attack patterns contained in the dataset. The Term Frequency–Inverse Document Frequency (TF–IDF) was used to represent the features in numerical form, making it ready for the feature selection and modeling. By integrating the normalized hyperbolic function, we improve the estimation of redundancy coefficients and effectively adapt the MIFS technique for early ransomware detection, i.e., before encryption takes place. Our proposed method, *e*MIFS, involves evaluating candidate features individually using the hyperbolic tangent function (tanh), which provides a suitable representation of the features’ relevance and redundancy. Our approach enhances the performance of existing MIFS techniques by considering the individual characteristics of features rather than relying solely on their collective properties. The experimental evaluation of the *e*MIFS method demonstrates its efficacy in detecting ransomware attacks at an early stage, providing a more robust and accurate ransomware detection model compared to traditional MIFS techniques. Moreover, our results indicate that the integration of the normalized hyperbolic function significantly improves the feature selection process and ultimately enhances ransomware early detection performance.

## 1. Introduction

The rapid increase in digital interconnectivity and reliance on technology has made cyber-attacks, especially malware, a significant global threat [1,2,3]. Malware, or malicious software, has evolved since the early 1970s, represented by various emerging types such as viruses, worms, Trojans, spyware, and ransomware [4,5,6]. Ransomware, which holds user data and files for ransom by denying access, gained popularity among attackers when enabling technologies such as Ransomware-as-a-Service (RaaS), internet, cryptography, and hard-to-trace digital currencies emerged [7,8]. These technologies allow even inexperienced attackers to create and distribute ransomware and receive payment without a significant risk–reward fear of being caught. Currently, it is unclear what percentage of attackers are ever brought to justice. Anecdotally, there are examples of circumstances where the ransom has been recovered (e.g., Colonial Pipeline).

Kaspersky reports that ransomware attacks are increasingly targeting businesses, with 30% of infections in 2019 affecting corporate users [9]. Financial losses due to ransomware attacks have been substantial, reaching billions of dollars worldwide in recent years. These attacks have surged in recent years, costing an average of USD 170,000 per attack [10]. Since 2020, several major ransomware attacks have occurred, including WannaCry, NotPetya, REvil, BlackByte, and LockBit 2.0, targeting various sectors such as healthcare, manufacturing, and government [11]. Such an escalation is attributed to several factors like the growing sophistication of ransomware, increased use of cloud computing, and the COVID-19 pandemic. Some notable cases include the following:

- Colonial Gas Pipeline attack in May 2021 caused gasoline shortages and price hikes. The JBS meatpacker attack in July 2021 led to a shutdown of operations in the US and Australia.
- Kaseya VSA software attack in December 2021 affected over 1500 businesses across 100 countries [12]. Victims of ransomware suffer not only from denied access to data but also from downtime costs, financial loss, and reputational damage.

### Ransomware Overview

Ransomware is typically characterized as either locker ransomware or crypto-ransomware based on severity. Crypto-ransomware attacks are particularly concerning, as they cannot be reversed even after removing the malware, often leaving victims with no choice but to pay the ransom for a decryption key [13]. To effectively protect digital assets, early detection of impending ransomware attacks is crucial before the encryption phase can take place [14]. Invariably, early detection can be achieved by observing and analyzing the processes running on a victim’s machine during the pre-encryption phase [15]. However, detecting ransomware in its early stages is very challenging due to insufficient data as well as the variability of attack patterns at this phase [16].

Consequentially, during the early stages of ransomware attacks, the small amount of data collected poses challenges for early detection and results in low detection accuracy [17,18]. Moreover, pre-encryption data lacks sufficient attack pattern characterization for the detection model to make accurate decisions while simultaneously avoiding the disruptive consequences of false positive courses of action. Prior studies have shown that feature selection techniques cannot identify the necessary crucial features that distinguish ransomware behaviors from normal process behaviors [15,16]. High dimensional features generated by feature extraction methods like *n*-gram can lead to overfitting, and many of these features are either too common, too specific, or redundant, ultimately degrading detection accuracy [19]. Redundant features may also cause the exclusion of informative features, which makes early detection even more challenging.

Several studies focusing on ransomware prevention and detection have indeed focused on understanding the characteristics of attack patterns, including the varied behaviors of ransomware [20]. These studies have explored state-of-the-art detection methodologies aimed at disrupting the escalating ransomware problem. Additionally, there has been a shift towards the development of machine learning-based detection systems, leveraging dynamic analysis and classification techniques to identify and prevent the constantly moving target of ransomware attacks [21]. Furthermore, the use of cryptographic algorithms and reinforcement learning has been proposed as a means to enhance ransomware detection and defense mechanisms [22,23]. In response to the increasing sophistication of ransomware attacks, researchers have introduced innovative approaches such as file-based ransomware detectors and self-configurable prevention techniques for the Internet of Medical Things (IoMT) [24,25]. Additionally, the development of early-stage detection systems based on pre-attack internal API calls has been explored as a means to mitigate ransomware attacks [26]. The use of game theory and multi-tier streaming analytics models has been investigated to enhance the detection of 0-day ransomware attacks using machine learning [27,28]. Likewise, the analysis of ransomware attack behaviors and the development of prevention mechanisms have been crucial in understanding the impact of ransomware, such as the LockBit 2.0 ransomware, and devising strategies to avoid such attacks [29]. Additionally, the use of predictive analysis and context-aware AI in IoT systems has been proposed to predict and detect ransomware penetration attempts in resource-constrained IoT environments [30].

Numerous different feature selection techniques are incorporated in ransomware and malware detection solutions to reduce data dimensionality and remove redundant features [31]. Redundancy and relevancy are the main factors that affect the performance of these techniques, which aim to filter out redundant and irrelevant features while retaining informative ones [32]. However, redundancy and relevancy are not always separate, and some relevant features may also be redundant. Therefore, a redundancy–relevant trade-off is needed during the selection process and is crucial for the feature selection technique to effectively manage this trade-off [33].

Information theory-based feature selection excels in balancing redundancy and relevancy without assuming a specific underlying data distribution, making it suitable for use in ransomware early detection where such distributions are hard to perceive [16,34]. However, this information theory-based method struggles with sparse and incomplete attack patterns, generating suboptimal feature sets due to the reliance on mutual information calculations. Consequently, incomplete data during the pre-encryption phase of ransomware attacks makes it difficult to identify common characteristics, leading to redundant and irrelevant features.

The current techniques that employ a redundancy coefficient calculation are not well-suited for early attack detection, as the collected data lacks sufficient attack patterns. This issue negatively impacts the MIFS goal function’s ability to accurately estimate the redundancy coefficient due to data sparsity that makes it difficult to discern the common characteristics of features in the selected list. As a result, an enhancement to the redundancy estimation mechanism within the MIFS goal function is necessary. Although some studies have tried to address this issue by individually comparing the candidate feature with each feature in the already-selected set, they assume that the redundancy increases linearly with the size of the selected set. This does not always hold, as the redundancy score of the features can vary and not necessarily be linear, especially with early pre-encryption data that contains incomplete attack patterns.

This paper aims to address this problem by proposing an enhanced redundancy estimation method for the enhanced Mutual Information Feature Selection (*e*MIFS) technique to improve the accuracy of ransomware detection. Rather than increasing the redundancy score linearly, our technique uses the normalized hyperbolic function that follows the S shape. Such a regime represents the change of the redundancy score for incomplete pre-encryption data. To this end, the contributions of this paper are three-fold.

- Propose an improved redundancy–relevancy trade-off technique for the goal function of the MIFS using the normalized hyperbolic function *e*MIFS.
- Integrate the improved *e*MIFS into the training phase of the ransomware early detection model.
- Conduct an experimental evaluation to measure the accuracy of our improved model and compare it with the existing solutions found in the literature.

## 2. Related Works

As mentioned above, ransomware attacks target various systems and networks, while studies on detecting these attacks can be categorized into data-centric and process-centric approaches [5,35]. Data-centric approaches monitor user data and files, raising alarms when detecting suspicious changes. However, they do not distinguish between changes made by benign programs and those by crypto-ransomware, leading to false positive alarms and ineffective early detection. Process-centric approaches monitor running processes’ behavior to discover suspicious patterns or computational resource usage but often rely on the entire runtime data or ad hoc events, which can lead to false alarms and/or be unsuitable for early detection. Both approaches have limitations in effectively detecting ransomware before the main sabotage phase, i.e., encryption takes place.

Several approaches attempt to detect crypto-ransomware early by using pre-encryption phase data to train machine learning algorithms [15,36,37,38]. Most of those studies rely on a set of features selected based on their relevancy estimation, which helps to select the most important features while reducing data dimensionality. In the study [16], the authors proposed a Redundancy Coefficient Gradual Upweighting (RCGU) technique to address the challenge of estimating feature significance in the early (pre-encryption) phase of crypto-ransomware attacks with insufficient attack patterns. The RCGU technique individually calculates redundancy between the candidate feature and each feature in the selected set, making it more effective in detecting redundancy with limited pre-encryption data. The RCGU approach eliminates the need for difficult-to-perceive common characteristics extraction and improves the redundancy–relevancy trade-off. One of the main contributions of this paper includes proposing the RCGU technique be incorporated with the MaxMin approximation technique. In this way, we thereby emphasize the importance of the redundancy term. We have conducted extensive experimental evaluations to demonstrate and validate the efficacy of the proposed combined techniques.

The study conducted by [15] proposed an Enhanced Maximum Relevance and Minimum Redundancy (EmRmR) algorithm for high-dimensional ransomware feature selection. The method involves a two-step feature reduction technique using API call-sequence refinement and feature redundancy. The approach refines the data based on the ransomware’s dynamic characteristics during execution and applies a dynamic programming technique to achieve maximum relevance and minimum redundancy. The EmRmR algorithm is compared with the original mRmR method in terms of running time and evaluations on different datasets. Additionally, the performance of five machine-learning classifiers trained on the refined system call sequences is evaluated to determine the proposed method’s effectiveness. As a result, the paper’s main contributions include introducing a refinement process for system call traces, proposing the EmRmR method to improve feature selection efficiency, and comparing their performance with the original mRmR results.

An automated wrapper-based feature selection method [27] used along with Particle Swarm Optimization (PSO) was suggested as a way to identify and categorize ransomware based on its behavior. The approach achieves the following: (i) eliminates the need for a predetermined number of input features, (ii) addresses feature selection in datasets with many dimensions by using a group-based strategy through PSO, and (iii) offers insights into the suitability of the chosen features for ransomware detection and classification. These are some of the method’s primary contributions. The study compared the performance and quantity of features selected from the proposed method with two other feature selection techniques: Variable-Length Particle Swarm Optimization (VarLenPSO) and Self-adaptive Particle Swarm Optimization (SaPSO).

The research conducted by [39] focuses on the development of robust features for ransomware detection systems to effectively handle concept drift, which can render current features ineffective. While machine learning classifiers have proven effective in detecting zero-day malware threats, zero-day ransomware detection remains a challenge. The study distinguishes between zero-day ransomware and truly evolved ransomware based on their behavioral patterns during execution. The paper contributes by analyzing the evolving behavioral characteristics of ransomware and proposing a feature selection architecture (FeSA) that provides an optimal feature set with promising performance under concept drift, maintaining a slower degradation rate in detection.

Malware visualization, automatic feature extraction, and classification were all provided by MalFCS [29], a malware categorization mechanism. The malware binaries are represented as entropy graphs by the framework, which uses convolutional neural networks with deep layers to extract features. It is evaluated against current techniques using the Microsoft and Malimg benchmark datasets. With precision rates of 0.997 and 1, respectively, MalFCS performs exceptionally well in classification and is more resilient to data imbalances than alternative methods. This is the first study to show that malware entropy graphs may be used to distinguish families of malware since convolutional neural networks with deep layers can be applied to them. By adding to current techniques, the suggested framework can make it more difficult for malware to avoid detection.

The framework [40] combined machine learning and blockchain technology to enhance malware detection accuracy for IoT devices. Machine learning was used for Android malware detection, creating a vast malware database. The trained model results were stored in a blockchain-based framework. The methodology first distinguished malware from benign applications and refined them using enhanced clustering methods, which calculated feature weights and iteratively reduced unnecessary features. A multi-feature Naive Bayes algorithm was proposed for malware classification, extracting vital characteristics and removing noisy objects. Permissioned blockchain was used to store de-tracked malware feature information, enabling efficient runtime detection. The framework could be directly applied to mobile devices and offered improved accuracy, robustness, and speed in detecting and classifying malware.

A malware classification method that uses pre-trained deep convolutional networks with features extracted from the grayscale images of malware binaries is described in a feature fusion-based strategy [31]. Once malware binaries have been converted into grayscale images, data augmentation techniques are applied to resolve data imbalances in the malware datasets. The best multimodal feature representation is then produced by merging deep convolutional neural network (DCNN) features and segmentation-based fractal texture analysis (SFTA) features into a single vector. This feature representation makes the malware classification model more reliable.

An Android malware static detection method using a Siamese Convolutional Neural Network (SCNN) was proposed by [41]. The main contributions included proposing an effective feature extraction method based on frequency in different applications, being the first to utilize Siamese CNN for Android malicious application detection, comparing distance calculation methods and finding Euclidean distance to be more effective for Siamese CNN, and proposing a benign and malicious mean center strategy [41], that can improve detection efficiency by calculating the distance from two mean centers for category judgment.

However, the lack of sufficient data during early attack phases affects the feature selection process, leading to increased redundant features, higher data dimensionality, and decreased detection accuracy. Our paper proposes an enhanced technique, which improves the feature selection process by making better redundancy–relevancy trade-offs, allowing for better estimation of feature significance and overcoming the insufficiency in attack patterns collected during early crypto-ransomware attack phases.

## 3. Preliminaries

Mutual information (MI) is a measure of how much information one variable provides about another. It can be used to evaluate the relationship between two variables, such as a feature and a target variable. By selecting features with high mutual information scores, we can reduce the dimensionality of the dataset while preserving the most relevant and informative features. This can lead to improved model performance, increased interpretability, and reduced computational cost. MI-based feature selection techniques are particularly useful when dealing with large datasets containing numerous features with varying degrees of relevance and redundancy.

Let us define two variables, *X* and *Y*, where *X* = {*x*_1, *x*_2, …, *x*_i} is an array of input features, and *Y* is the vector of output labels. Thus, MI measures how much information two variables share. Concretely, it is calculated using Equation (1), where *I(X;Y)* is the MI between variables *X* and *Y*, and *P(X)*, *P(Y)*, and *P(X,Y)* are the marginal and joint distributions of X and Y.

(1) $$I\left(X,Y\right)=\sum_{y\epsilon Y}\sum_{x\epsilon X}\left(p\left(x,y\right)\right)log\frac{p\left(x,y\right)}{p(x)p(y)}$$

The general formula for MI-based feature selection *J(Xk)* is shown in Equation (2). It includes the MI between a candidate feature and the class label and the conditional MI between the candidate feature and the selected feature set, given the class label. The coefficients in the equation have values between 0 and 1.

(2) $$J\left(X_{k}\right)=I\left(X_{k},Y\right)-\beta \sum_{x_{j}\epsilon s}I\left(X_{j},X_{k}\right)+\gamma \sum_{x_{j}\epsilon s}I(X_{j},X_{k}|Y)$$

Equation (2) can be decomposed into a relevancy term and a redundancy term. The relevancy term (the 1st expression) measures the amount of information that a candidate feature provides about the class label. The redundancy term (the 2nd and 3rd expressions) measures the amount of information that a candidate feature shares with the selected feature set. Typically, the coefficients *β* and *γ* are calculated based on the following equation:

(3) $$\beta =\gamma =\frac{1}{\left|S\right|}$$

where S denotes the number of selected features. Therefore, when the number of selected features increases, the value of these coefficients decreases. There are two types of MI-based feature selection techniques: those that consider only the relevancy term and those that consider both the relevancy term and the redundancy term. The first type of technique, such as Mutual Information Feature Selection (MIFS) and Minimum Redundancy Maximum Relevance (mRMR), only considers the amount of information that a candidate feature provides about the class label. The second type of technique, such as Joint Mutual Information (JMI), considers both the amount of information that a candidate feature provides about the class label and the amount of information that it shares with the selected feature set.

The performance of MI-based feature selection techniques depends on the way that redundancy is calculated. The coefficients (*β* and *γ*) in Equation (2) play a crucial role in the relevancy–redundancy trade-off, which determines the significance of a feature. Smaller values of the marginal redundancy coefficient (*β*) decrease the effect of redundancy, increasing the significance of a feature. Smaller values of the conditional redundancy coefficient (*γ*) also decrease the significance of a feature.

## 4. Methodology

In this methodology section, we outline the systematic approach and techniques employed to address the research contribution to achieve the objectives of this study. A comprehensive description is herein provided of the following: (i) data collection and pre-processing, (ii) feature selection, and (iii) model development processes, as well as (iv) evaluation metrics used to assess the performance of the proposed solution. By presenting a clear and detailed explanation of the methods used, we ensure that the study is transparent, replicable, and can be built upon by other researchers in the field. The following sections delve into each aspect of the methodology, highlighting the rationale behind the chosen techniques and their respective contributions to the overall study.

### 4.1. Data Collection and Pre-Processing

Data are gathered in the Cuckoo Sandbox, where ransomware samples are run in a controlled environment and stored in trace files. Then, the data underwent various data pre-processing activities, which included operations like noise removal, missing data imputation, and normalization, which were carried out to get the data ready for modeling. As it guarantees that the data is clear and devoid of information that is unrelated or misleading, eliminating noise is a crucial stage in the pre-processing of data. Measurement mistakes, missing values, and outliers are a few examples of how noise can be introduced. These can all have a detrimental effect on the MIFS detection model’s performance and lead to conclusions that are unreliable or wrong. To find and remove outliers in each dataset feature, a filter based on the statistical mean and standard deviation was used in this procedure.

#### *e*MIFS’s Architecture

**Figure 1:**
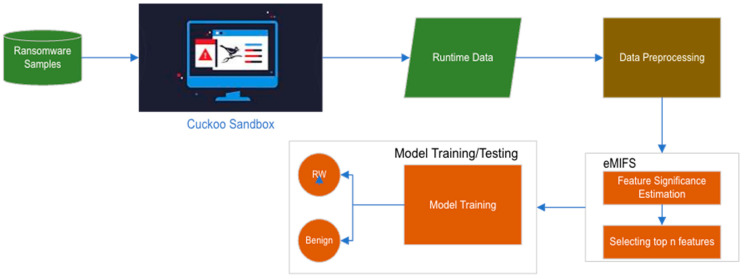
The architectural design of the ransomware detection model with the proposed *e*MIFS.

Figure 1 shows the architectural design of our *e*MIFS ransomware detection model. The model comprises several components starting with ransomware samples run inside the Cuckoo Sandbox. The runtime data are then stored in trace files, which undergo data pre-processing to remove the noise and vectorization to convert data into a numerical form. The vectorization was carried out using Term Frequency Inverse Document Frequency, which prepares the data for the next step and makes it suitable for modeling. Pre-processing also involves data standardization by which the data are scaled between 0 and 1. The processed data are then fed into the *e*MIFS to select the top n desired features. These features are used to train the detection model. The model is composed of a machine-learning classifier that checks the new data instances against its learned knowledgebase and recommends the decision as to whether it is ransomware or benign.

Scaling attribute values to a range between 0 and 1 is known as normalization. Its goal is to stop the machine learning algorithm from training by placing an undue emphasis on qualities with wider value ranges. By doing this, normalization aids in balancing the impact of big values on the training procedure, which may otherwise cause the algorithm to give some attributes more excessive weight than others. Normalization also lessens the chance of overfitting and helps the algorithm converge more quickly. In this way, normalization guarantees that no feature is given preference by the model and offers a more realistic depiction of the connections between the various features in the dataset. Equation (4) below is therefore employed to standardize the data.

(4) $$X_{norm}=(X-X_{min})/(X_{max}-X_{min})$$

Equation (4) produces a normalized value (X_norm_) for each entry (i.e., entity) within the dataset. This is accomplished by deducting the minimal value (X_min_) from X and dividing the resulting number by the dataset’s range (X_max_–X_min_). The feature’s scale is made consistent throughout the dataset thanks to this normalizing method, which guarantees that the value of X is scaled to the range between 0 and 1.

### 4.2. A Normalized Hyperbolic Redundancy Coefficient Upweighting

This section describes our suggested method for estimating feature redundancy, which we name Normalized Hyperbolic Redundancy Coefficient Upweighting (NH-RCU). Determining the degree of confidence in the redundancy term is largely dependent on the coefficient *β*. We provide the NH-RCU strategy, which uses Equation (6) to evaluate redundancy, in contrast to the existing MIFS method, which uses Equation (5) to determine *β*.

(5) $$\beta =\frac{1}{\left|S\right|}$$

(6) $$\beta =0.5*(1+\tan\left(\frac{S}{F}\right))$$

where F denotes the original feature’s vector. When new features are introduced to the selected set, the NH-RCU algorithm slowly increases the weights rather than updating the value of *β* linearly. This technique guarantees the preservation of common traits among the selected features. The small size that the selected set S starts with is reflected by the initial setting of the *β* value to 0. Using the tanh function (6), *β* steadily climbs towards 1 as the set S expands. In Equation (6), the tangent was used to refine β to provide a distinct advantage in feature selection when dealing with sparse or limited datasets. The rapid change in the function’s output for small changes in input offers a sensitive mechanism to differentiate between the features with marginal differences in mutual information values. This sensitivity is particularly beneficial in early detection scenarios, where the distinction between relevant and irrelevant features must be made with high precision despite the paucity of data. The number of required features are used as a stopping criteria.

### 4.3. NHRCU-Based Mutual Information Feature Selection

**Figure 2:**
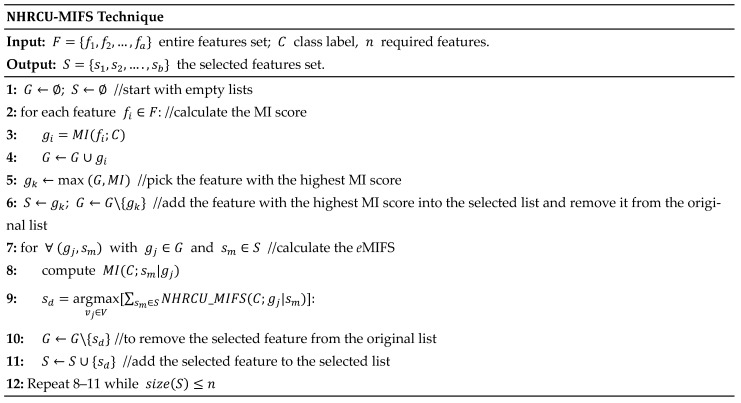
The pseudocode for the NHRCU-MIFS technique.

The goal of the *e*MIFS (also referred to as NHRCU-MIFS) is to choose the features that are most relevant to the class label. Each feature in the first feature set has its mutual information calculated as part of the process. Next, a new set of features is introduced to which the feature with the highest mutual information is attached. Until the required number of features is obtained, this process is repeated (refer to Equation (7)). Next, the features in the new set are ordered according to how relevant they are to the class label. The features that rank higher are kept, while the lower features are eliminated. The *e*MIFS technique improves our machine learning model accuracy and minimizes data noise by concentrating on the most important features. It is a versatile technique that can benefit various applications by improving model performance through effective feature selection. Figure 2 shows the pseudocode of the proposed NHRCU-MIFS technique.

(7) $$NHRCU\_MIFS\left(x_{k}\right)=MI\left(x_{k},y\right)-0.5*(1+\tan\left(\frac{S}{F}\right))\left(\sum_{s_{j}\epsilon S}I\left(x_{k},x_{j}\right)\right)$$

### 4.4. The Dataset

The study collected ransomware PE files from Virusshare, a public repository, which included 39,378 ransomware samples from families like CryptoWall, Petya, and WannaCry. Additionally, 9732 benign applications were downloaded from informer.com. The files were executed in the Cuckoo Sandbox virtual platform for analysis. Cuckoo Sandbox is an open-source framework that analyzes malware in a controlled and isolated environment, tracing API calls and network traffic and providing comprehensive reports in JSON format. Following the same procedure as those specified above in the related works section, the study used the Cuckoo Sandbox to run ransomware samples and collect runtime data. The sandbox submits the sample to a guest machine imitating a victim’s device and captures the runtime data, including API calls. The guest machine is then restored to a clean state for the next analysis, ensuring no influence from any previous infections. The JSON files generated were used to create a dataset, from which features were extracted and selected before training the detection model.

### 4.5. Experimental Environment and Evaluation Metrics

The *e*MIFS method was implemented and evaluated using a variety of software and tools, including Python 3.8, SK-feature, TensorFlow 2.14, Keras 2.14, Scikit Learn 1.3, and Numpy 1.26. The data preparation, algorithm execution, and analysis of results were carried out on a computer with an Intel**^®^** Core™ i7-4790 CPU @ 3.60 GHz and 16 GB of RAM. The effectiveness of the *e*MIFS approach was evaluated using accuracy. The approximation error of the model was measured using its false positive and false negative rates. The detection accuracy was calculated using Equation (8). The results showed that the *e*MIFS method was able to achieve high accuracy on the dataset. The *e*MIFS method is a promising approach for improving the accuracy of the detection model. It is able to select features that are most relevant to the class label, which helps to reduce the noise in the data and improve the accuracy of the detection model.

(8) $$ACC=\frac{TP+TN}{TP+TN+FP+FN}$$

where *TP*, *TN*, *FP*, and *FN* denote the true positive, true negative, false positive, and false negative, respectively.

## 5. Results, Discussion, and Analysis

For the first encryption stage of the ransomware lifecycle, the most informative features were selected using the *e*MIFS method. The following feature sets, with varying amounts of features, were tested: 5, 10, 15, 20, 25, 30, 35, 40, 45, and 50. Each of these feature sets was used to train a variety of machine learning classifiers, including Long Short-Term Memory (LSTM), Random Forests (RF), Support Vector Machines (SVM), Logistic Regression (LR), and Deep Belief Networks (DBN). Using a 10-fold cross-validation method, the classifiers’ accuracy was evaluated at various feature quantities. The dataset was split into training and testing sets. The classifiers’ accuracy in classification was then assessed using the testing set.

### 5.1. Maximizing Feature Discriminative Accuracy

Table 1 provides the accuracy results for each classifier per feature set. Notably, we observe that the accuracy of the classifiers increases as the number of features grows until a certain limit, after which the accuracy stops increasing or declines. This indicates that the top features selected by the proposed *e*MIFS were discriminative enough to identify the attack patterns even in the absence of sufficient data. This claim is also supported by the decline that happens after adding more features caused by the effect of overfitting beyond the feature discriminative power. For example, LR accuracy increases to 0.919 when the feature size reaches 30. When the number of features surpasses 30, LR’s accuracy fluctuates between 0.913 and 0.917. Similarly, the SVM accuracy increases to 0.93 as the number of features grows to 20, but the accuracy then declines as the number of features continues to increase.

Likewise, the RF accuracy rises to 0.934 as the number of features reaches 20, but the accuracy falls when the number of features exceeds 25. Moreover, we observed that the use of deep learning maintains the increase in accuracy as the number of features grows. This phenomenon is evident in the DBN, where the accuracy continues to increase from 0.927 to 0.962 as the number of features ranges from 5 to 45. Similarly, LSTM accuracy increases from 0.93 to 0.967 as the number of features grows from 5 to 45. However, the increase in accuracy becomes less steep when the number of features surpasses 35 for both deep learning DBN and LSTM models. Such accuracy values indicate that the top 25 features selected by *e*MIFS carry sufficient information about the early ransomware attack patterns. This is attributed to the ability of the normalized hyperbolic function to control the redundancy calculation of the candidate feature. Instead of increasing the redundancy score linearly, the NHRCU increases it according to the tanh function that is bound between 0 and 1. Such a regime is more realistic as the redundancy does not follow a constantly increasing rate.

### 5.2. Improving NH-RCU Accuracy via MIFS Integration

**Figure 3:**
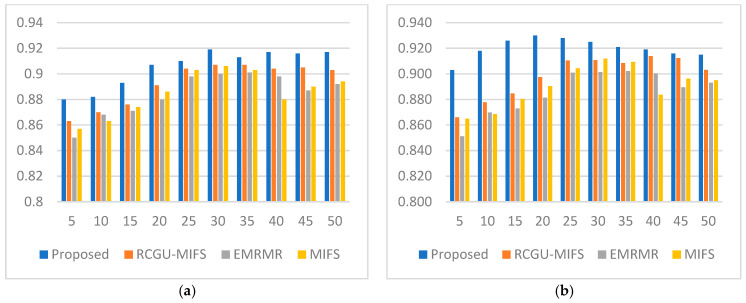

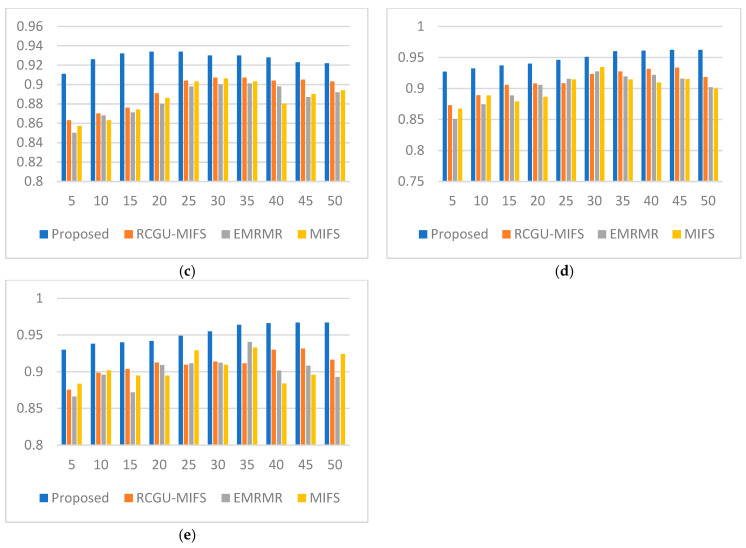
These graphs compare the accuracy of our NH-RCU with the comparable works (i.e., RCGU-MIFS, EMRMR, and MIFS) employing a number of classifiers, including (**a**) LR, (**b**) SVM, (**c**) RF, (**d**) DBN, and (**e**) LSTM.

To show the efficacy of the proposed NH-RCU technique when integrated with MIFS, the results are compared with the RCGU-MIFS [16], EMRMR [15], and MIFS [42] feature selection techniques. The same machine learning classifiers (LR, SVM, RF, DBN, and LSTM) are used to measure accuracy using different sizes of feature sets ranging from 5 to 50 and incremented by 5 features between each two subsequent sets (sets with a higher number of features, e.g., 10, 15, 20, … etc.). Therefore, classification accuracy is used as the measurement of classification performance. Figure 3a–e shows the comparison results between the proposed NH-RCU and the related techniques. Based on these comparison results, the proposed NH-RCU outperforms RCGU, EMRMR, and MIFS.

Consider Figure 3a–e, which show that the proposed NH-RCU technique achieved detection accuracies higher than the RCGU_MIFS of [16] for all selected set sizes. Our proposed technique also outperformed the accuracy of *e*MIFS and MIFS. This is attributed to the ability of the proposed NH-RCU function in estimating the redundancy score of the candidate features better than the existing methods in the cases of insufficient data. This demonstrates that *e*MIFS has the ability to overcome circumstances to represent the change of redundancy score when the number of features increases in the already-selected set. This indicates that the proposed techniques were able to perceive information from the features better than those captured by existing feature selection techniques.

The comparison results shown in Figure 3a–e indicate how performance improvements are achieved by increasing the number of features up to a certain limit, as described above. Notably, when the limit is reached, the accuracy tails off and potentially declines. This effectively defines the limit empirically due to the effect of the redundancy score that grows when the number of features increases. Predictably, and consequently, this supports our assumption that redundancy can be measured between the individual features instead of the common characteristics of the feature set.

Another reason for accuracy curtailment is the well-known effect of overfitting. The accuracy (including false positive/negative rates) of ransomware deterrence models suffers when the number of features increases beyond a specific threshold. Again, this can be attributed to the increase in the redundancy score that features carry when more features are added to the selected set. Classical machine learning classifiers like LR, SVM, and RF experience accuracy declines more typically after a certain limit of feature numbers. However, such a decline does not happen for deep learning algorithms like DBN and LSTM due to their ability to perceive hidden characteristics in the features that could not be perceived by classical machine learning.

### 5.3. Perceiving Hidden Feature Characteristics

There is a limit to which the number of selected features contributes to improving accuracy. This limit varies based on the number of features selected within each run. For example, the maximum accuracy was obtained when training the LR model with 30 features, whereas the maximum accuracy for SVM was obtained training with 25 features. The problem of overfitting, which is brought on by adding features and increasing the number of training epochs, is responsible for the decline in accuracy (or the barely noticeable improvement) that occurs as the number of features in the detection model’s training increases.

The dimensionality of the data rises with the number of features. Large data dimensionality negatively impacts the detection model’s accuracy. When working with data that does not exhibit enough early-stage ransomware behavior, the dimensionality issue gets even worse. Moreover, there is a greater chance of overfitting when the model’s training incorporates a larger number of features. As a result, the classifier’s loss function is unable to distinguish between false and actual patterns with sufficient accuracy. Consequently, after a certain number of features, accuracy begins to decline or show no further improvement.

### 5.4. Comparing Characteristic Measures of eMIFS Robustness

**Figure 4:**
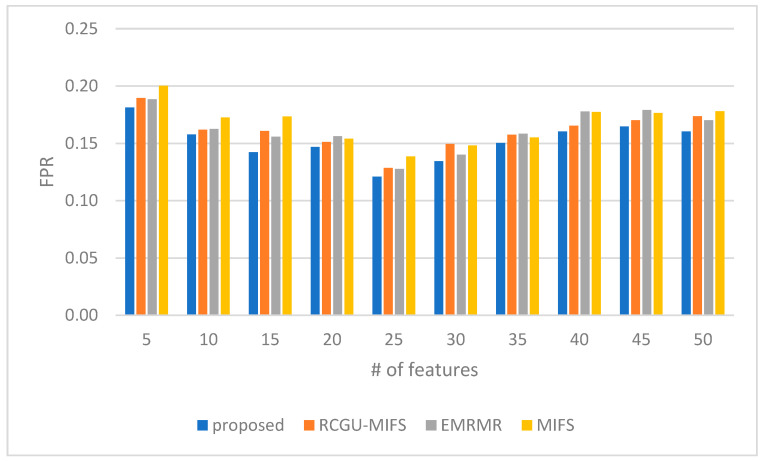
A comparison of the False Positive Rate (FPR) of our NH-RCU with comparable works (RCGU-MIFS, EMRMR, and MIFS).

We also conducted a comparative evaluation between the proposed and related techniques in terms of False Positive Rate (FPR), Detection Rate (also called false positive rate), and F1 score. The comparison of false positive rates (FPR) between the proposed feature selection technique and related techniques is shown in Figure 4. It reveals nuanced performance across different feature counts. Initially, for a low feature count (5), the proposed technique shows a lower FPR of 0.18 compared to those observed in the related techniques, indicating a minor but notable efficiency in reducing false alarms with minimal feature sets. As the number of features increases to 10 and 15, the proposed technique continues to exhibit lower or equal FPRs (0.16 and 0.14, respectively), suggesting its effectiveness in maintaining lower false positives even as the feature count increases. Notably, at 25 features, the proposed technique achieves its lowest FPR of 0.12, surpassing the related techniques and highlighting its superior capability in minimizing false positives with an optimal number of features.

However, as the feature count increases beyond 30, the FPR for the proposed technique slightly rises but remains competitive, indicating a consistent performance across a range of feature sizes. Specifically, at higher feature counts (40 to 50), the proposed and related techniques converge towards similar FPRs, with the proposed method maintaining a better performance compared to its counterparts. This pattern suggests that while the proposed technique is particularly effective at lower to mid-range feature counts in minimizing false positives, its advantage becomes less at higher feature counts. However, it still remains an efficient choice for reducing false positives.

**Figure 5:**
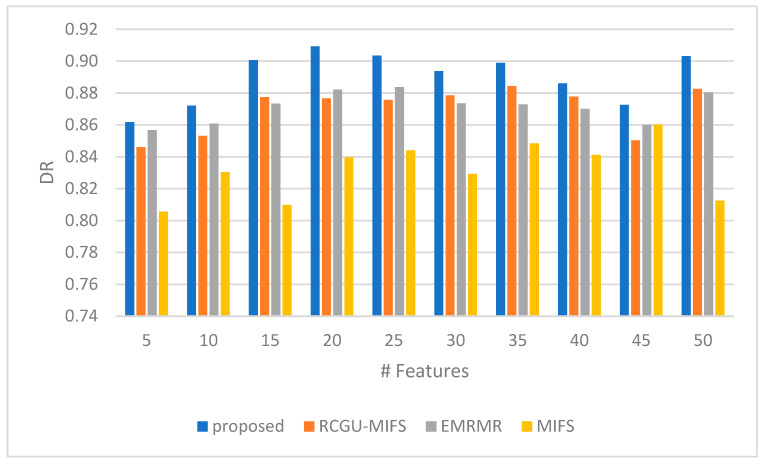
A comparison of the Detection Rate (DR) of our NH-RCU with comparable works (RCGU-MIFS, EMRMR, and MIFS).

The detection rate shown in Figure 5, a comparison between the proposed feature selection technique and related techniques, highlights the effectiveness of the proposed method across various feature counts. Initially, with 5 features, the proposed technique starts with a detection rate of 0.86. As the feature count grows, at 15 and 20 features, the proposed technique shows its peak performance with detection rates of 0.90 and 0.91, respectively, indicating its efficiency in leveraging larger feature sets for improved detection. Notably, it outperforms all related techniques at these points, suggesting its superior ability to utilize additional information for better threat identification. The detection rates for the proposed technique slightly fluctuate but remain high across different feature counts, highlighting its consistency in maintaining high detection rates.

Towards the higher end of the feature spectrum (35 to 50), the proposed technique’s detection rates exhibit slight variations but generally remain better, with a notable return to a higher rate of 0.90 at 50 features. This demonstrates the technique’s adaptability and effectiveness across a wide range of feature counts. Although the detection rates of the related techniques improve or remain steady in some cases, the proposed technique consistently presents a better performance.

**Figure 6:**
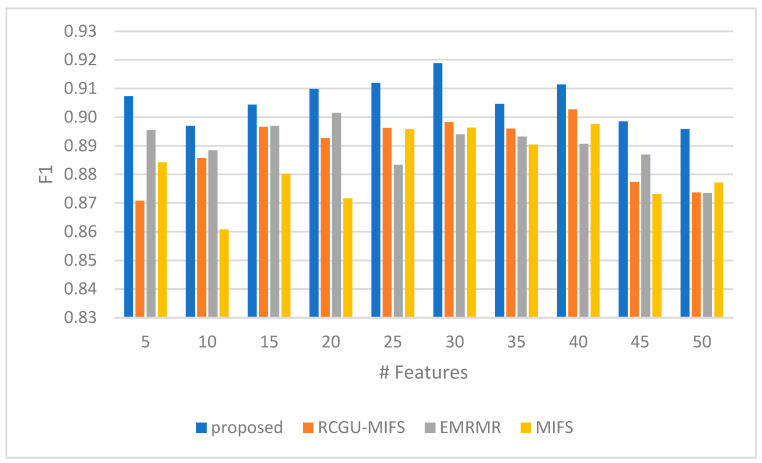
A comparison of the F1 score of our NH-RCU with comparable works (RCGU-MIFS, EMRMR, and MIFS).

The F1 in Figure 6 measures a comparison between the proposed feature selection technique and demonstrates the proposed method’s consistent and effective performance in balancing precision and recall across various feature counts. Starting with five features, the proposed technique exhibits a better F1 measure of 0.91, outperforming the related techniques. This trend is maintained as the number of features increases. At the mid-range of feature counts (15 to 25), the proposed technique maintains an F1 measure of 0.90 to 0.91, indicating its robustness in utilizing an optimal number of features for maximum effectiveness. Notably, at 30 features, the proposed method achieves its peak F1 measure of 0.92, showing its capability to maximize the harmonic mean of precision and recall with a moderately large feature set. This is particularly noteworthy as it outperforms the related techniques, underscoring the proposed technique’s ability to achieve a balance between false positives and false negatives.

As the feature count further increases to 40 and beyond, the proposed technique’s F1 measure remains high, demonstrating its consistent performance and adaptability across a wide range of feature counts. Although the related techniques exhibit improvements or maintain stable performance in some cases, the proposed technique shows competitive F1 values, highlighting its effectiveness in the proposed feature selection ransomware analysis. This consistent performance across different feature counts shows the ability of the proposed technique to achieve optimal detection capabilities with an emphasis on precision and recall balance.

### 5.5. Outlook and Significance of These Results

Future research in ransomware detection can focus on bridging the gap between the high resource demands of AI-based methods and the efficiency of non-AI-based techniques. AI solutions, known for their adaptability and precision in identifying complex threats, are often prohibitive for resource-constrained settings due to their computational intensity. A key research trajectory involves refining AI algorithms to be more resource-efficient without sacrificing performance through methods like model pruning and the development of streamlined neural networks. Concurrently, enhancing non-AI methods to improve their adaptability and incorporating AI insights could offer immediate, low-resource solutions. The development of hybrid models that merge the strengths of both AI and non-AI approaches promises a versatile and scalable cybersecurity framework adaptable to various computational constraints. Addressing this disparity in resource consumption is crucial for practical ransomware detection, ensuring comprehensive protection across diverse organizational sizes and capacities in the face of rapidly advancing ransomware attacks.

## 6. Conclusions and Future Research

In this paper, we have presented an *enhanced* MIFS, i.e., *e*MIFS. This technique incorporates a normalized hyperbolic function for ransomware early detection models. Our approach addresses the challenges associated with the insufficiency of attack patterns in the dataset and the difficulty in perceiving common characteristics among features.

By individually evaluating candidate features using the hyperbolic tangent function (tanh), we have improved the redundancy coefficient estimation and adapted the MIFS technique for early ransomware detection. We have named this improved technique *e*MIFS to emphasize an *enhanced* ransomware detection capability because of the typical sparsity of characteristic behavior data, especially when early detection is crucial.

The experimental results have demonstrated the efficacy of our proposed method in detecting ransomware attacks at an early stage, outperforming traditional MIFS techniques. The integration of the normalized hyperbolic function has significantly improved the feature selection process, leading to a tailored, more robust, and accurate ransomware detection model. The improvement has been demonstrated by the results shown in Figure 3a–e. It suggests that the *e*MIFS has better performance characteristics compared to results from either of the EMRMR and MIFS model techniques proposed in prior studies.

The use of the tangent function in *e*MIFS for calculating the Beta (β) coefficient, however, may cause several challenges, such as volatility in the feature selection phase due to unbounded outputs, which can lead to overfitting in varied or noisy data. Regularization techniques are needed to maintain model generalizability. Additionally, the tangent function can increase computational complexity and resource demands, particularly with larger datasets, which may affect scalability and require more efficient algorithms or computing power for practical applications.

Future research directions include exploring alternative functions and approaches to further enhance feature selection and redundancy estimation in the context of ransomware detection. Additionally, it would be beneficial to investigate the applicability of the proposed method to other types of cybersecurity threats and intrusion detection scenarios. Ultimately, our work contributes to the ongoing efforts to develop more effective and efficient methods for ransomware early detection, helping to mitigate the ever-growing risks posed by these malicious attacks.

## Figures and Tables

**Table 1:** Experimental results of the *e*MIFS with different sizes of feature sets used to train several classifiers.

# Features	LR	SVM	RF	DBN	LSTM
5	0.88	0.903	0.911	0.927	0.93
10	0.882	0.918	0.926	0.932	0.938
15	0.893	0.926	0.932	0.937	0.94
20	0.907	0.93	0.934	0.94	0.942
25	0.91	0.928	0.934	0.946	0.949
30	0.919	0.925	0.93	0.951	0.955
35	0.913	0.921	0.93	0.96	0.964
40	0.917	0.919	0.928	0.961	0.966
45	0.916	0.916	0.923	0.962	0.967
50	0.917	0.915	0.922	0.962	0.967

## Data Availability

Data is unavailable due to privacy.
